# Novel digenic inheritance of *PCDH15* and *USH1G* underlies profound non-syndromic hearing impairment

**DOI:** 10.1186/s12881-018-0618-5

**Published:** 2018-07-20

**Authors:** Isabelle Schrauwen, Imen Chakchouk, Anushree Acharya, Khurram Liaqat, Irfanullah, University of Washington Center for Mendelian Genomics, Michael J. Bamshad, Suzanne M. Leal, Deborah A. Nickerson, Peter Anderson, Marcus Annable, Elizabeth E. Blue, Kati J. Buckingham, Imen Chakchouk, Jennifer Chin, Jessica X. Chong, Rodolfo Cornejo, Colleen P. Davis, Christopher Frazar, Martha Horike-Pyne, Gail P. Jarvik, Eric Johanson, Ashley N. Kang, Tom Kolar, Stephanie A. Krauter, Colby T. Marvin, Sean McGee, Daniel J. McGoldrick, Karynne Patterson, Sam W. Phillips, Jessica Pijoan, Matthew A. Richardson, Peggy D. Robertson, Isabelle Schrauwen, Krystal Slattery, Kathryn M. Shively, Joshua D. Smith, Monica Tackett, Alice E. Tattersall, Marc Wegener, Jeffrey M. Weiss, Marsha M. Wheeler, Qian Yi, Di Zhang, Deborah A. Nickerson, Michael J. Bamshad, Khadim Shah, Wasim Ahmad, Suzanne M. Leal

**Affiliations:** 1 Center for Statistical Genetics, Department of Molecular and Human Genetics, Baylor College of Medicine, One Baylor Plaza 700D, Houston, TX 77030 USA; 2 Department of Biotechnology, Faculty of Biological Sciences, Quaid-i-Azam University, Islamabad, Pakistan; 3 Department of Biochemistry, Faculty of Biological Sciences, Quaid-i-Azam University, Islamabad, Pakistan; 4 Department of Genome Sciences, University of Washington, Seattle, Washington, USA; 5 Department of Pediatrics, University of Washington, Seattle, Washington, USA

**Keywords:** Digenic inheritance, Hearing impairment, Deafness, *PCDH15*, *USH1G*

## Abstract

**Background:**

Digenic inheritance is the simplest model of oligenic disease. It can be observed when there is a strong epistatic interaction between two loci. For both syndromic and non-syndromic hearing impairment, several forms of digenic inheritance have been reported.

**Methods:**

We performed exome sequencing in a Pakistani family with profound non-syndromic hereditary hearing impairment to identify the genetic cause of disease.

**Results:**

We found that this family displays digenic inheritance for two *trans* heterozygous missense mutations, one in *PCDH15* [p.(Arg1034His)] and another in *USH1G* [p.(Asp365Asn)]. Both of these genes are known to cause autosomal recessive non-syndromic hearing impairment and Usher syndrome. The protein products of *PCDH15* and *USH1G* function together at the stereocilia tips in the hair cells and are necessary for proper mechanotransduction. Epistasis between Pcdh15 and Ush1G has been previously reported in digenic heterozygous mice. The digenic mice displayed a significant decrease in hearing compared to age-matched heterozygous animals. Until now no human examples have been reported.

**Conclusions:**

The discovery of novel digenic inheritance mechanisms in hereditary hearing impairment will aid in understanding the interaction between defective proteins and further define inner ear function and its interactome.

**Electronic supplementary material:**

The online version of this article (10.1186/s12881-018-0618-5) contains supplementary material, which is available to authorized users.

## Background

Over the last decade, genetic studies have taught us that there is a continuous spectrum of genetic influences between monogenic and oligogenic diseases. The simplest model of multifactorial inheritance is digenic, where in its original definition, two loci are necessary to express or extremely modify the severity of a phenotype. Compared to monogenic disease inheritance, digenic inheritance does not follow Mendelian segregation and is probably underdiagnosed due to the difficulty in verifying true digenic effects. However, several convincing cases of digenic inheritance have been found in genetically heterogeneous disorders including hearing impairment (HI) [1–4]. These findings encouraged researchers when analyzing exome and genome sequence data to consider variants in related genes or similar pathways that fit a digenic disease model as candidates, which has led to additional promising reports [5–9].

Several putative digenic recessive interactions causing non-syndromic (NS) HI and syndromic HI, e.g. Usher and Pendred syndromes have been described [1–9]. Digenic *GJB2* (Cx26) and *GJB6* (Cx30) heterozygous variants are an often observed cause of HI in humans [4, 5]. A 309-kb deletion, also referred to as del (GJB6-D13S1830), which involves *GJB6*, causes HI in the homozygous state, or in the compound heterozygous state with a large variety of *GJB2* mutations. However, this example should be considered as monogenic *GJB2* autosomal recessive NSHI and not truly digenic in its underlying molecular nature, since the *GJB6* deletion inactivates *GJB2* [10, 11], which is its neighboring gene on chromosome 13.

There are several examples of true digenic inheritance for HI. For example, digenic inheritance of *CDH23* and *PCDH15* is well established [1], and has been shown to cause age-related HI in mice, and Usher Syndrome Type I in humans. Both proteins interact closely and are crucial for the normal organization of the stereocilia bundle. Digenic heterozygous mice showed degeneration of the stereocilia and a base-apex loss of hair cells and spiral ganglion cells [1]. Other described digenic cases include *SLC26A4* and *FOXI* [2], which causes Pendred syndrome or HI associated with enlarged vestibular aqueducts (EVA) in humans or EVA in the mouse mutant, and *SLC26A4* and *KCNJ10* [3], which have been observed to cause HI and EVA in humans. In addition, some putative digenic inheritances have been suggested but still require further evidence or need to be replicated, such as *GJB2* and *TMPRSS3* [7] and *MYO7A* and *PCDH15* [8], amongst others.

For HI, dominant ‘digenic’ additive effects of two genes have also been described, which leads to a more severe hearing loss than the effect of a single variant. For example, for a Swedish family, an additive effect of linked loci *DFNA2* and *DFNA11,* resulted in a more severe phenotype for which the causative variants and genes have yet to be identified [12].

Digenic inheritance can refer to different scenarios [13, 14], and there is currently no clear consensus regarding the definition of digenic inheritance. The most commonly used definition, requires two loci for expression or extreme modification of the severity of a similar phenotype. There is a thin line between the digenic modification definition and genetic modifiers, as both are often used in a similar context.

The Digenic Diseases Database (DIDA) [13] classifies digenic cases into two classes which are simplifications of the original definitions provided by Schäffer [15]: 1) The first class is referred to as the ‘true digenic’ class, i.e. variants at both loci are required for expression of the disease, and neither variant alone displays a phenotype. 2) The second class is a composite class as it includes different possibilities, such as Mendelian variants plus modifiers that vary the phenotype, or dual molecular diagnoses, wherein Mendelian variants at each of the two loci segregate independently and results in a combination of both phenotypes [13]. However, there are a spectrum of scenarios possible that can blur these defined borders [14]. In *OMIM* (Online Mendelian Inheritance in Man), digenic inheritance is classified into two categories: Digenic dominant inheritance is defined as heterozygous mutations in two genes, while digenic recessive inheritance signifies a homozygous or compound heterozygous mutation in one gene and a heterozygous mutation in a second gene.

The digenic inheritance described in this article entails a true digenic model, in which two *trans* heterozygous mutations in two genes (on different chromosomes) whose protein products function closely together at the stereocilia tips in the hair cells (*PCDH15* and *USH1G*) are required for the expression of a phenotype.

## Methods

The study was approved by the Institutional Review Boards of the Quaid-i-Azam University and the Baylor College of Medicine and Affiliated Hospitals (H-17566). Written informed consent was obtained from all participating members.

**Fig. 1:**
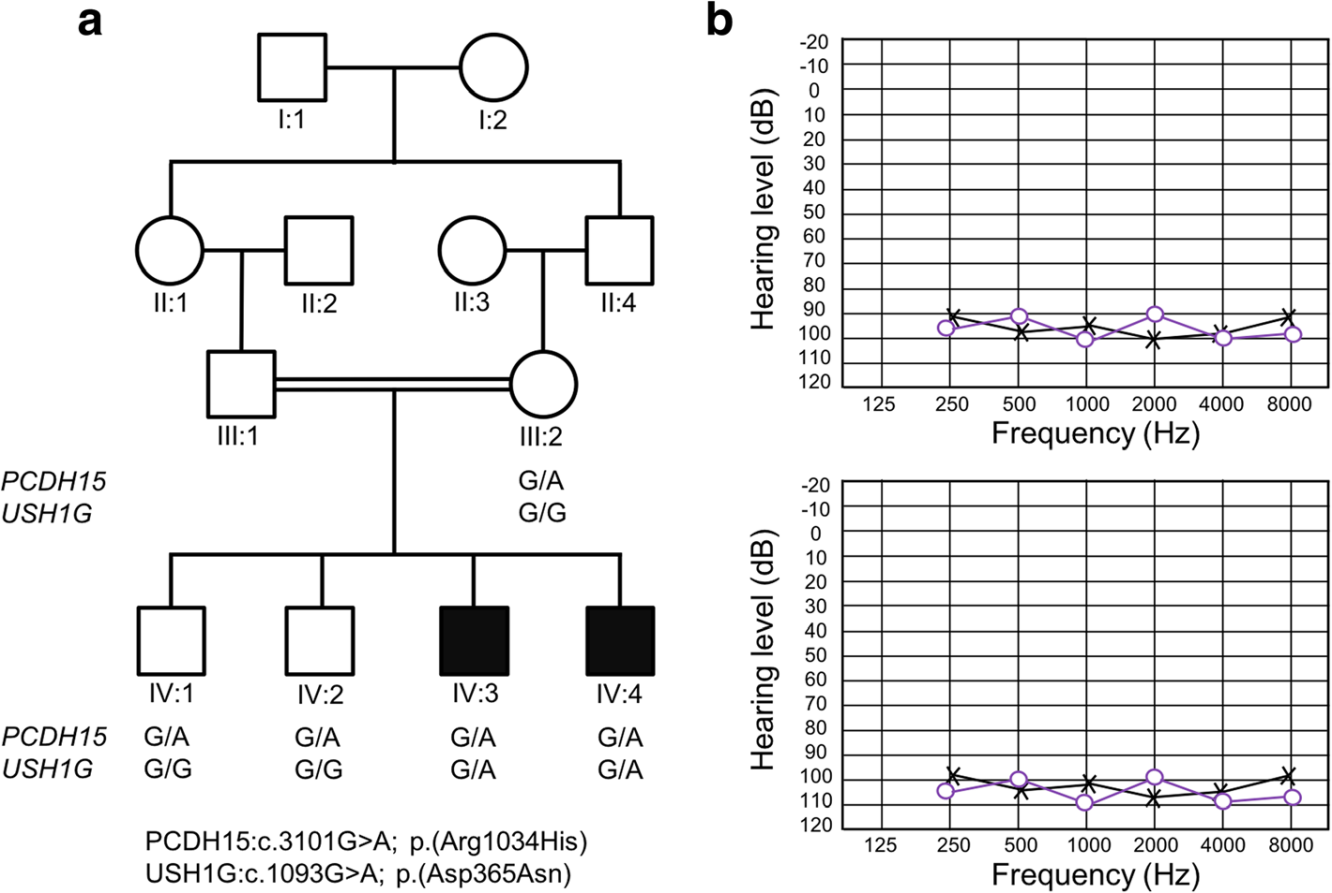
Pedigree drawing for family 4667 and audiograms for the affected family members. Panel **a** Pedigree drawing displaying family members with NSHI as filled symbols and unaffected family members as clear symbols. Males are represented by squares and females by circles. For the three unaffected and two affected family members genotypes for the *PCDH15* variant NM_033056:c.3101G > A and *USH1G* variant NM_173477:c.1093G > A are shown under each family member and demonstrate digenic inheritance. The DNA sample from Individual IV:4 was exome sequenced. Panel **b** Audiograms for affected family members IV:3 (top) and IV:4 (bottom). Pure-tone audiometry was performed between 250 and 8000 Hz and x represents the results for the left ear and o for the right ear. Affected individual IV:3 was 34 years old, and affected individual IV:4 was 22 years old at the time of pure-tone audiometry and physical examination

DNA samples were collected from five family members of a consanguineous family with hereditary non-syndromic hearing loss (Family 4667; Fig. 1a) from the Khyber Pakhtunkhwa province in Pakistan. These samples include DNA from two affected siblings (IV:3 and IV:4), two unaffected siblings (IV:1 and IV:2) and their mother (III:2) (Fig. 1a).

Genomic DNA was extracted from peripheral blood using a phenol chloroform procedure [16]. Exomic libraries were prepared from one affected individual (IV:4) with the Roche NimbleGen SeqCap EZ Human Exome Library v.2.0 (~ 37 Mb target), following the manufacturer’s protocol. Sequencing was performed by 70 bp paired-end sequencing on a HiSeq2500/4000 instrument (Illumina Inc., San Diego, CA, USA). Reads were aligned to the Human genome (Hg19/GRC37) using the Burrows-Wheeler transform (BWA-MEM), PCR duplicates were removed with Picard MarkDuplicates, and indel realignment was performed (GATK IndelRealigner). Single nucleotide polymorphisms (SNP) s and small insertions/deletions (Indels) variants were recalibrated with BaseRecalibrator and called jointly with HaplotypeCaller (GATK), annotated with dbNSFP and ANNOVAR for further filtering and interpretation [17]. Copy number variants (CNVs) were called using CoNIFER [18] and XHMM [19].

Variants were further filtered based on location (coding region and splice region +/− 12 bp), and frequency [minor allele frequency (MAF) Genome Aggregation Database (gnomAD) < 0.005 in all populations]. Variants with a predicted damaging functional effect were identified (e.g., splice-site, non-synonymous, nonsense, etc.), and conservation scores (e.g., PhastCons, GERP), and the Combined Annotation Dependent Depletion (CADD) score were evaluated prior to testing for segregation within the pedigree. We selected both heterozygous and homozygous variants for segregation testing in the pedigree, assuming several modes of inheritance possible in this pedigree: autosomal recessive (homozygous or compound heterozygous), X-linked, germline mosaicism or parental mosaicism, and digenic.

Sanger sequencing was used to validate variants and verify segregation with the HI phenotype in the family. Primers surrounding region of interest were designed using primer3 software [20]. PCR amplified products were treated with ExoSAP-IT™ PCR Product Cleanup Reagent (ThermoFisher Scientific, Sugerland, TX) and sequenced using the BigDye terminator v3.1 cycle sequencing kit (Applied Biosystems, Foster City, CA) on an ABI 3130 Genetic Analyzer (Applied Biosystems, Foster City, CA).

## Results

### Clinical evaluation

Pure-tone audiometry showed bilateral profound HI in both affected persons (Fig. 1b). An external eye exam, visual acuity and ophthalmoscopy, showed no vision problems. Other causes of HI, including infections, trauma and ototoxic medications were evaluated and excluded. Tandem gait and Romberg tests were performed to evaluate for gross vestibular deficits. No vestibular problems were identified. Careful physical examinations revealed no other problems in addition to HI in the family members, supporting that the HI is non-syndromic.

### Exome and Sanger sequencing

Exome sequencing revealed several variants of interest (Additional file 1: Table S1), which were all tested for segregation by performing Sanger sequencing using DNA from all available family members. None of the variants in genes previously associated with HI segregated with the HI phenotype with the exception of the *PCDH15* [GRCh37/hg19; chr10:55719513C > T; NM_033056: c.3101G > A; p.(Arg1034His)] and *USH1G* [GRCh37/hg19; chr17:72915838C > T; NM_173477:c.1093G > A; p.(Asp365Asn)] variants which displayed digenic inheritance (Fig. 1a).

The *PCDH15* variant [NM_033056: c.3101G > A; p.(Arg1034His)] has a CADD score of 23.9, is predicted damaging according to MutationTaster, and is conserved amongst species (GERP++ RS 4.53 and PhyloP20way 0.892). The variant is not present in the gnomAD database of 123,136 exomes and 15,496 whole-genomes of unrelated individuals, which includes 15,391 South Asian exomes [21]. In addition, the variant is not present in the Greater Middle East (GME) Variome Project that contains 1111 unrelated individuals from the Greater Middle East, including 168 Iranian and Pakistani individuals [22]. The variant was not observed in 81 in-house Pakistani exomes which had other Mendelian Traits but not NSHI or syndromic HI. This variant, in the homozygous state, was previously been described as pathogenic in an Iranian family with NSHI [23].

The *USH1G* [NM_173477:c.1093G > A; p.(Asp365Asn); rs538983393] variant has a CADD score of 22.9, is predicted damaging according to MutationTaster, and is conserved amongst species (GERP++ RS 4.53 and PhyloP20way 1.000). It has a low frequency in gnomAD (3.3 × 10^− 5^ overall; 2.3 × 10^− 4^ South Asian), with no homozygotes reported, and is not present in the GME Variome Project nor our in-house exomes. The *PCDH15* and *USH1G* variants are available in ClinVar (accession SCV000608345) [24].

Additionally for family 4667, we identified a heterozygous variant in *CDH23* [NM_022124:c.C2263T:p.(His755Tyr); rs181255269] via exome sequencing. It was tested for segregation and is present in a heterozygous state in all individuals with an available DNA sample (III: 2, IV: 1, IV: 2, IV: 3 and IV: 4). Although this variant was originally suggested to be pathogenic [25], based upon recent evidence in ClinVar, and a high population frequency in certain populations (2.2% MAF in the Turkish Peninsula [22]; Additional file 1: Table S1), this variant is likely benign. This variant also does not fit a digenic inheritance model with known digenic partner *PCDH15* in this family.

To find any other potentially missed pathogenic variants in this family, we examined the BAM files for individual IV:4 using Integrative Genomics Viewer (IGV2.3.97) to try to detect any variants that were not called and/or regions with no reads or low read depth (<= 8× coverage). All low and/or uncovered exonic and splice regions of *USH1G* and *PCDH15* were Sanger sequenced, and no additional variants were found. We also performed a CNV analysis on the exome data, and only one heterozygous deletion was called in the sequenced exome of individual IV: 4 by both CoNIFER and XHMM (GRCh37/hg19; chr13:100511115–100,915,087). This region does not contain any known HI genes. Additionally, no other CNVs in this region have been reported in the Database of Genomic Variants (DVG) associated with any disease [26].

## Discussion

Hair cells of the inner ear are mechanosensors for the detection of sound and balance/movement. At the apical surface of each hair cell is its mechanically sensitive organelle, the hair bundle, which consists of dozens of stereocilia. Mechanotransduction channels are located near stereociliary tips and open or close on deflection of the stereocilia. Tip-links stretch from the tips of stereocilia in the short and middle rows to the sides of neighboring, taller stereocilia. These Tip-links on stereocilia are made of cdh23 and pcdh15 [27]. In the Ames waltzer mice, recessive mutations of *Pcdh15* cause deafness due to disorganized stereocilia bundles and degeneration of inner ear neuroepithelia [28].

Sans, the protein coded by *Ush1g*, interacts with the cytoplasmic domains of cdh23 and pcdh15 in vitro and is absent from the hair bundle in mice defective for either of the two cadherins [27]. Sans (*Ush1g)* localizes mainly to the tips of short- and middle-row stereocilia in vivo, and plays a critical role in the maintenance of molecular complex at the lower end of the tip-link [27]. Thus, Sans locates at stereocilia tips, near the location of Pcdh15. In *Ush1g*^−/−^ mice, the cohesion of stereocilia is also disrupted, and both the amplitude and the sensitivity of the transduction currents are reduced [27]. Interaction between USH1G and PCDH15 is further demonstrated in digenic heterozygous mice. +/*Pcdh15*^*av-3J*^ +/*Ush1g*^*js*^ double heterozygous mice display hearing loss, with highly significant elevated auditory brainstem response (ABR) thresholds at 3–4 months [29], suggesting Pcdh15-Ush1g epistasis [29].

In the traditional definition, epistasis describes the interaction of two or more genetic loci, which can substantially modify disease severity or result in an entirely new phenotype. In the literature within and between different fields, there are contradictions in the definitions and interpretations of epistasis [30]. Adopting the original definition of epistasis, a non-linear interaction, we describe a family where the hearing impaired members carry *trans* heterozygous variants in *PCDH15* and *USH1G* and have profound HI and single variant carriers have normal hearing (Fig. 1). We cannot, confirm epistasis in vitro, i.e. biochemical epistasis [31]. We hypothesize that the biochemical function of their network is severely affected by these two variants and results in a profound HI, because both proteins function together at the stereocilia tips in the hair cells and are necessary for proper mechanotransduction. Since each gene separately is known to cause autosomal recessive HI, reduced activity/functioning of both proteins in the same close interacting network is a likely disease model.

## Conclusions

In this study, we suggest epistasis between PCDH15 and USH1G in humans, through the study of a consanguineous family with profound hereditary HI, segregating a heterozygous and predicted damaging mutation in both *PCDH15* and *USH1G* (Fig. 1). Digenic inheritance of hearing impairment in mice and humans suggest that the proteins interact or perform co-dependent functions in hair cells. The study of digenic diseases can help us understand more about the complex interaction within the inner ear and is an initial step towards the understanding of more complex oligogenic diseases, such as age-related hearing loss.

## Additional file

Additional file 1: Supplementary data associated with this manuscript consists of **Table S1.** Variants of interest identified by exome sequencing. This list includes variants in this study that were tested for segregation. Annotations and population frequencies are listed. (XLSX 12 kb).

## Abbreviations

ABR: Auditory Brainstem Response
CADD: Combined Annotation Dependent Depletion
CNV: Copy Number Variant
CoNIFER: Copy number inference from exome reads
DGV: Database of Genomic Variants
DIDA: Digenic Diseases Database
EVA: Enlarged vestibular aqueducts
ExAC: Exome Aggregation Consortium
GATK: Genome Analysis Toolkit
GERP: Genomic Evolutionary Rate Profiling
GME: Greater Middle East Variome Project
GnomAD: Genome Aggregation Database
HI: Hearing Impairment
MAF: Minor Allele Frequency
NSHI: Non-syndromic Hearing Impairment
OMIM: Online Mendelian Inheritance of Man
PCR: Polymerase Chain Reaction
XHMM: eXome-Hidden Markov Model
